# Healthcare Resource Utilization and Treatment Costs for Blastic Plasmacytoid Dendritic Cell Neoplasm: A PETHEMA Study

**DOI:** 10.3390/cancers17172844

**Published:** 2025-08-29

**Authors:** Antonio Solana-Altabella, Irene Navarro-Vicente, Eduardo Rodríguez-Arbolí, Victor Noriega, Josefina Serrano, Teresa Bernal, Vicente Carrasco-Baraja, Raimundo Garcia-Boyero, Carmen Olivier Cornacchia, Lorenzo Algarra, Eduardo López-Briz, Armando Mena-Durán, Jackeline Solano-Tovar, Carmen Botella-Prieto, Sara Sánchez-Sánchez, Juan Miguel Bergua-Burgues, Pilar Lloret-Madrid, Mario Rodenas-Rovira, Blanca Boluda, Isabel Cano-Ferri, Evelyn Acuña-Cruz, Rebeca Rodríguez-Veiga, Laura Torres-Miñana, María Centelles-Oria, José Luis Poveda-Andrés, David Martínez-Cuadrón, Pau Montesinos

**Affiliations:** 1 Pharmacy Department, Hospital Universitari i Politècnic La Fe, 46026 Valencia, Spain; solana_ant@gva.es (A.S.-A.); lopez_edubri@gva.es (E.L.-B.); rodenas_marrov@gva.es (M.R.-R.); maria_centelles@iislafe.es (M.C.-O.); poveda_josand@gva.es (J.L.P.-A.); 2 Accredited Research Group on Hematology, Instituto de Investigación Sanitaria La Fe (IISLAFE), 46026 Valencia, Spain; irene_navarro@iislafe.es (I.N.-V.); pilar_lloret@iislafe.es (P.L.-M.); boluda_bla@gva.es (B.B.); isabel_cano@iislafe.es (I.C.-F.); evelyn_acuna@iislafe.es (E.A.-C.); rebeca_rodriguez@iislafe.es (R.R.-V.); laura_torres@iislafe.es (L.T.-M.); martinez_davcua@gva.es (D.M.-C.); 3 Accredited Research Group on Pharmacy, Instituto de Investigación Sanitaria La Fe (IISLAFE), 46026 Valencia, Spain; 4 Hematology Department, Hospital Universitari i Politècnic La Fe, 46026 Valencia, Spain; 5 Hematology Department, Hospital Universitario Virgen del Rocío, 41013 Sevilla, Spain; edurodarb@gmail.com; 6 Instituto de Biomedicina de Sevilla (IBIS/CSIC), 41009 Sevilla, Spain; 7 Faculty of Medicine, Universidad de Sevilla, 41004 Sevilla, Spain; 8 Hematology Department, Hospital Universitario A Coruña, 15006 A Coruña, Spain; victor.noriega.concepcion@sergas.es; 9 Hematology Department, Hospital Universitario Reina Sofía, Instituto Maimónides de Investigación Biomédica de Córdoba (IMIBIC), Universidad de Córdoba (UCO), 14004 Córdoba, Spain; josefina.serrano@iname.com; 10 Hematology Department, Hospital Universitario Central de Asturias, 33011 Oviedo, Spain; bernaldelcastillo@gmail.com; 11 Hematology Department, Hospital Universitario Royo Villanova, 50015 Zaragoza, Spain; vcarrascob@aragon.es; 12 Hematology Department, Hospital General Universitari de Castelló, 12004 Castellón de la Plana, Spain; garcia_rai@gva.es; 13 Hematology Department, Complejo Asistencial de Segovia, 40002 Segovia, Spain; colivierco@gmail.com; 14 Hematology Department, Complejo Hospitalario Universitario de Albacete, 02008 Albacete, Spain; hemato78@hotmail.com; 15 Hematology Department, Hospital General Universitario de Valencia, 46014 Valencia, Spain; mena_arm@gva.es; 16 Hematology Department, Complejo Asistencial de Palencia, 34005 Palencia, Spain; jackelinest@saludcastillayleon.es; 17 Hematology Department, Hospital General Universitario Dr. Balmis, 03010 Alicante, Spain; carmenbotellaprieto@gmail.com; 18 Hematology Department, Hospital Marina Baixa, 03570 Villajoyosa, Spain; sanchez_sarsan@gva.es; 19 Hematology Department, Hospital San Pedro de Alcántara, 10003 Cáceres, Spain; jmberguaburg@gmail.com

**Keywords:** healthcare costs, blastic plasmacytoid dendritic cell neoplasm, hospitalization, reimbursement, healthcare resource utilization

## Abstract

Background/Objectives: Information on the economic impact and healthcare resource utilization (HCRU) associated with Blastic Plasmacytoid Dendritic Cell Neoplasm (BPDCN) is lacking. Methods: A retrospective review of medical records identified patients diagnosed with BPDCN between 2009 and 2023. Data on outpatient resource use, reimbursement, frequency and duration of hospitalizations, and transfusion burden were collected from diagnosis to death or last follow-up. Results: A total of 38 patients with a median age of 66 years were included. Conventional intensive chemotherapy (CHT) regimens were initially administered to 58% of patients, while targeted therapies were used in 37% of cases. The ratio of total days hospitalized to the overall follow-up period was 17%, with a total of 182 hospitalizations (average of 5 per patient; mean duration 20 days). The mean total reimbursement was EUR 109,104 per patient, primarily attributed to hospital admissions (EUR 89,158; EUR 18,118 per hospitalization). Hospitalizations before or without any allogeneic hematopoietic stem cell transplant (alloHSCT) accounted for 70% of total admissions, with an average overall cost of EUR 50,285 per patient (EUR 12,427 per hospitalization). AlloHSCT-period hospitalizations occurred in 18 patients, with a mean total reimbursement of EUR 122,497 per patient and EUR 30,464 per hospitalization. Conclusions: The active treatment of BPDCN imposes a high economic burden and extensive HCRU. Comparative pharmacoeconomic studies evaluating the cost-effectiveness of new therapies for BPDCN are needed to identify patient subgroups that may benefit most from these treatments.

## Simple Summary

Blastic Plasmacytoid Dendritic Cell Neoplasm (BPDCN) is a rare and aggressive blood cancer that often requires intensive medical care. In this study, we looked at how much it actually costs to treat patients with this disease and how often they use hospital services. The average cost per patient is over EUR 100,000, and hospital stays make up a big part of that. These patients spend a lot of time in hospital, which shows just how demanding this condition is on the healthcare system. These data are especially important when dealing with rare diseases, where resources are limited and treatment choices can be complex.

## 1. Introduction

Blastic Plasmacytoid Dendritic Cell Neoplasm (BPDCN) is an extremely rare and aggressive hematologic malignancy characterized by the proliferation of malignant plasmacytoid dendritic cells. BPDCN predominantly affects older adults and is associated with poor prognosis, with a median overall survival (OS) ranging from 8 to 16 months for patients receiving conventional therapies, including non-intensive schemes [1,2]. Clinically, BPDCN often presents skin lesions, lymphadenopathy, bone marrow infiltration, and peripheral blood involvement, contributing to its complex management [2].

Historically, treatment options for BPDCN have included CHT regimens, typically adapted from acute lymphoblastic leukemia (ALL) or acute myeloid leukemia (AML) protocols. However, these approaches have shown limited long-term efficacy and are often associated with significant toxicity. The approval of the CD123-targeted therapy tagraxofusp has marked an advancement in the treatment landscape, demonstrated, in a prospectively designed pivotal trial, by high response rates with a manageable safety profile including a lack of prolonged myelosuppression [2,3,4]. Despite this new therapy demonstrating clinical benefits, its high cost raises concerns about its affordability and widespread use [5], especially in healthcare systems with limited resources; even so, its different toxicity profile may result in reduced use of healthcare resources.

This study aims to evaluate the baseline healthcare resource utilization (HCRU) and costs associated with managing BPDCN by establishing a detailed understanding of the economic burden of BPDCN under standard care approaches. The present study was conducted by the Spanish Program for Hematology Treatments (PETHEMA) group, a national scientific consortium specializing in the treatment of hematological malignancies and the development of clinical trials in Spain. PETHEMA maintains a retrospective registry spanning more than 20 years, including clinical data from more than 25,000 patients with acute leukemias.

## 2. Materials and Methods

### 2.1. Patients and Study Design

Adult (≥18 years) patients diagnosed with BPDCN between 2009 and 2023 registered in the multicenter PETHEMA group database (NCT02607059) with available HCRU information were identified. Patients eligible for inclusion in the study were adults with at least one therapeutic scheme (intensive or not intensive).

This was a multicenter, retrospective pharmacoeconomic study. The study protocol was approved by the local Clinical Research Ethics Committee, following the principles outlined in the Declaration of Helsinki.

Individual patient data were collected from the date of BPDCN diagnosis until death or the last follow-up. The index date was defined as the first date on which the BPDCN diagnosis was documented in the patient’s clinical records. The cut-off date for data analysis was set as December 2024.

The CHT period was defined as the interval from the index date to loss to follow-up, the initiation of allogeneic hematopoietic stem cell transplantation (alloHSCT), or death. The alloHSCT period was defined as the time from hospital admission for alloHSCT to loss to follow-up or death.

### 2.2. Objectives

The primary objective of the study was to describe outpatient resource utilization, reimbursement, hospitalization frequency and duration, and transfusion burden. Secondary objectives focused on characterizing BPDCN patients regarding their demographic, clinical, and treatment characteristics at induction. HCRU specific to BPDCN was analyzed in a selected cohort, examining the frequency, type, and duration of hospitalizations, reasons for admission, and associated hospitalization reimbursements. Additionally, outpatient resources such as hospital day visits, clinic visits, and transfusions were evaluated. These variables were analyzed across both the CHT and alloHSCT periods. In addition, the subgroup of patients treated with anti-CD123 therapy, the only approved treatment for BPDCN, was specifically analyzed. Notably, multiple reasons for admission could be recorded for each hospitalization.

### 2.3. Calculation of Hospital Reimbursement

**Figure 1:**
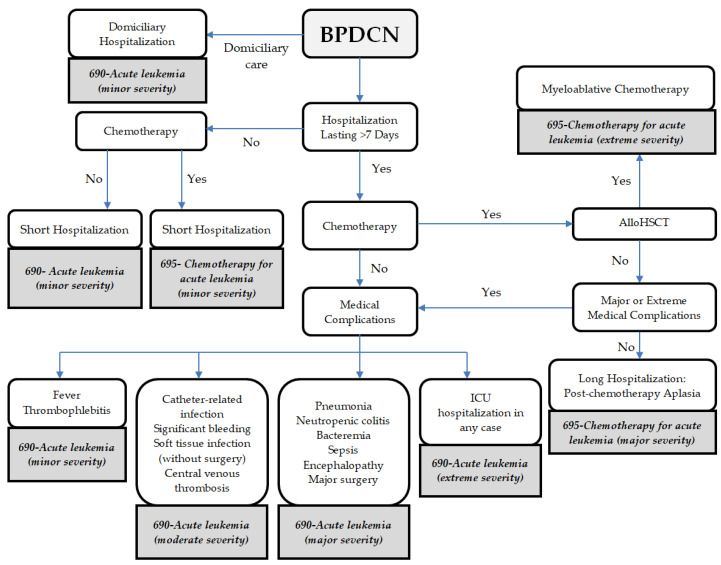
Decision algorithm for assignment of Diagnosis-Related Group (DRG) code for hospitalization episodes. *AlloHSCT—*allogeneic hematology steam transplantation; *BPDCN—*Blastic Plasmacytoid Dendritic Cell Neoplasm; *ICU*—Intensive Care Unit.

We used the 2023 national DRG assignment system (Table S1). The national Diagnosis-Related Group (DRG) list provides information on the amount of money reimbursed to the hospital and the average length of stay for each DRG code. Reimbursement for CHT, and other active treatments, does not need to be added to the costs because it is included in the reimbursement for the assigned DRG code. Reimbursement followed the algorithm for hematology hospitalizations at the center. The reimbursement for outpatient care was EUR 231 for each outpatient visit and EUR 262 for each day’s stay at a day hospital; these values were based on the mean day hospital unit costs, calculated using costs from all Spanish regions [6,7]. DRG codes (2023) and corresponding reimbursements were assigned with only one DRG code assigned for each hospitalization, as we described in Figure 1. For alloHSCT the DRG code *695-Extreme severity* was assigned and associated with a reimbursement of EUR 44.849 plus EUR 691 for each day beyond 40 days, and outpatient care after alloHSCT was EUR 691 [8,9].

### 2.4. Statistical Analyses

Based on the experience managing this rare disease, a target sample size of 40 patients was determined to ensure adequate statistical power. All patients who met the predefined inclusion criteria were included in the analysis. Quantitative variables were presented as means with standard deviations (SD) and medians with interquartile ranges (IQR). Categorical variables were described as frequencies and percentages. Subgroup analyses for the CHT and alloHSCT periods were conducted using negative binomial regression to assess differences in the number of hospital admissions, inpatient days, external consultation and day hospital visits, and associated costs, depending on the duration of exposure during each period.

Statistical analyses were conducted using Stata software 14.2.

## 3. Results

### 3.1. Patients

**Figure 2:**
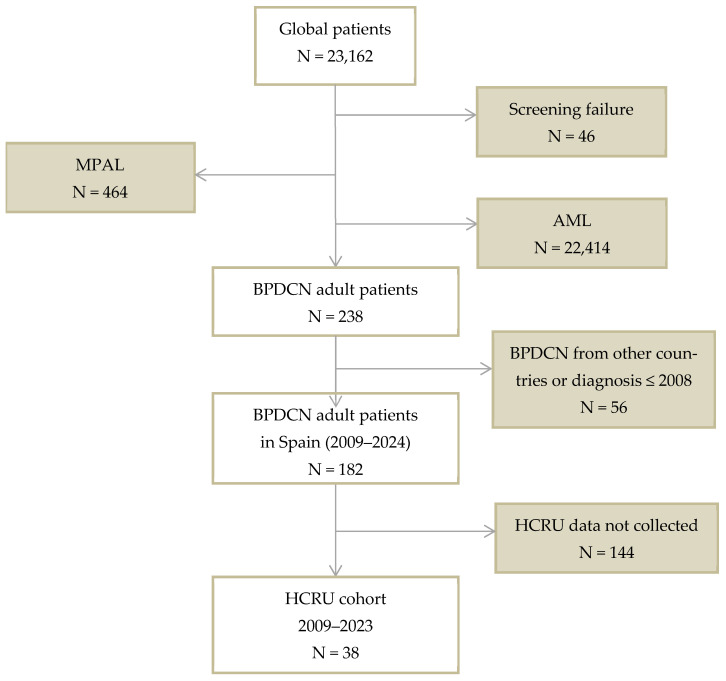
Flowchart diagram (patient enrolment). *AML*—Acute Myeloid Leukemia; *BPDCN*—Blastic Plasmacytoid Dendritic Cell Neoplasm; *HCRU*—Healthcare Resource Utilization; *MPAL*—Mixed-Phenotype Acute Leukemia.

A total of 182 patients diagnosed with BPDCN met the eligibility criteria for inclusion in the study, of which 38 had complete data on HCRU and hospitalization records (Figure 2). A patient was considered complete when all hospitalization information was available, including reasons for admission, along with outpatient consultations and hospital day visits, as well as transfusions administered. The median age of the cohort was 61 years, with a predominance of male patients (84%) (Table 1). The median follow-up period was 322 days, corresponding to a total of 21,913 days of follow-up or exposure for the entire cohort. Throughout the study, there were 187 hospital admissions, accounting for a total of 3618 inpatient days. The inpatient life vs. outpatient life ratio was 0.17. Patient recruitment was skewed toward more recent years, with the year of diagnosis for 21 patients (55%) occurring in 2020 or later (Figure S1). Although patients in the 2020–2023 period showed higher total healthcare costs and longer cumulative inpatient stays compared to those diagnosed between 2008 and 2019 (mean 110 vs. 77 days; EUR 116,967 vs. EUR 98,294), these differences were not statistically significant (*p* = 0.582 and *p* = 0.311, respectively).

Response to induction therapy resulted in 23 patients (60%) achieving complete remission (CR) or incomplete CR (CRi), 12 patients (32%) showing partial remission or resistance, and 3 patients (8%) experiencing induction-related mortality at any point during the induction phase, as detailed in Table S2.

For this part of the analysis, we evaluated HCRU across all study periods, from the index date to the last follow-up. Patients receiving BPDCN therapy had mean hospitalization duration of 95 days per patient, with an average overall reimbursement of EUR 89,158 per patient. Table 2 presents reimbursement and HCRU throughout the study period. By the end of the study, 25 patients (66%) had died.

### 3.2. Hospitalizations and Reimbursement During the Chemotherapy Period

In the first-line setting, 22 patients (58%) received intensive treatment regimens, while 16 patients (42%) received non-intensive regimens. Notably, two patients did not require any hospitalization, and one patient remained hospitalization-free prior to undergoing alloHSCT. The mean duration of the CHT period was 230 days (SD 150), during which a total of 128 hospitalizations occurred, with an average duration of 16 days per hospitalization. Each patient experienced an average of three hospitalizations (SD 2) (Table 3). During this period, the ratio of hospital stay to total treatment time was 31%. The mean total cost per patient was EUR 50,285 (SD EUR 28,408), with the majority attributed to hospitalization expenses (EUR 41,858; 83%), corresponding to a reimbursement of EUR 12,427 per hospitalization. Additionally, patients had a mean of 19 (SD 16) outpatient visits and 18 (SD 23) day hospital visits.

HCRU during the CHT period was similar between patients who received tagraxofusp as first-line treatment and those who did not (Tables S3 and S4). However, the mean total cost was higher in the first-line tagraxofusp group: EUR 56,903 (SD EUR 13,947) versus EUR 49,044 (SD EUR 30,361).

### 3.3. Hospitalizations and Reimbursement for Patients Who Underwent Allogeneic Hematopoietic Stem Cell Transplantation

A total of 18 patients underwent alloHSCT (Table 4). Throughout the alloHSCT period, there were 59 inpatient hospitalizations, with a mean length of stay of 27 days, comparable to the duration observed during the CHT treatment period. However, the reimbursement for inpatient hospitalizations during the alloHSCT period was higher, averaging EUR 30,464. Patients spent an average of 28% of the alloHSCT period hospitalized, with hospitalizations associated with a mean reimbursement of EUR 99,855 per patient and a total reimbursement of EUR 122,497 (SD EUR 88,738) (Table 4). By the end of the alloHSCT period, eight patients (44% of the subgroup) had died. No statistically significant differences were observed in HCRU or reimbursement based on the duration of exposure between the CHT and alloHSCT periods.

### 3.4. Hospitalizations and Reimbursement for Patients with Tagraxofusp as First-Line Treatment

A total of six patients received tagraxofusp (anti-CD123 therapy) as first-line treatment (Table S5). These patients accounted for 33 inpatient hospitalizations, with a mean length of stay of 15 days, which was shorter than that observed in the overall study population (Table 2). The average reimbursement per inpatient hospitalization was EUR 15,185. On average, patients spent 30% of the treatment period hospitalized. Overall, 50% of the patients in this subgroup underwent alloHSCT at some point during their treatment. Hospital admissions were associated with a mean reimbursement of EUR 80,986 per patient, resulting in a total inpatient reimbursement of EUR 96,683 (SD EUR 47,279) (Table S5).

### 3.5. Reasons for Hospitalizations

The most frequent primary reasons for hospitalization were the administration of chemotherapy (34%), fever (16%), alloHSCT (7%), and neutropenia (7%) (Figure S2). Admissions with a frequency of less than four were not analyzed separately and were categorized as “Other” (Figure S2).

## 4. Discussion

This multicenter study demonstrates that the current standard treatment for BPDCN is associated with prolonged hospitalizations and substantial healthcare costs. Following a BPDCN diagnosis, adult patients spend almost one-fifth of their remaining lifetime hospitalized, primarily due to CHT administration or severe complications. These hospitalizations contribute significantly to the overall costs associated with DRGs. Notably, studies evaluating the economic burden of BPDCN are nonexistent, and those analyzing the costs of AML are relatively limited in the literature.

Our research was conducted on a relatively small, unselected cohort of “real-world” BPDCN patients from the PETHEMA registry. Despite the sample size, our findings HCRU and associated costs can be cautiously extrapolated to the Spanish healthcare setting. We identified that hospitalizations are the primary driver of BPDCN treatment costs. Other relevant healthcare resource utilizations included transfusions, hospitalization and day hospital visits, and outpatient care, all of which also generated notable costs.

Due to the rarity of BPDCN, no specific data on HCRU are currently available. Therefore, comparisons can only be made using HCRU data from other hematologic malignancies, although such comparisons should be interpreted with caution given the differences in treatment approaches and disease prognosis. When comparing our findings with previous cost analyses in AML, the overall expenses observed in our cohort (EUR 108,293) were comparable to those reported in a Spanish relapsed/refractory AML cohort (EUR 109,104) [9]. However, HCRU during first-line treatment was not assessed, and the analysis was conducted in the aftermath of post-COVID inflation. In contrast, they were significantly lower than those observed in a United States (US) study on relapsed/refractory AML ($439,104) [10]. These discrepancies regarding the two studies may be attributable to differences in the methodology, as our study applied pre-defined DRG-based reimbursement rates, while the US study utilized individualized claims data. Additionally, the disparities could be influenced by the differences in per capita income between Spain and the US, where healthcare costs in Spain are primarily covered by a non-profit public health system, potentially leading to an underestimation of actual expenditures.

Interestingly, our results align with the perception of a potentially more efficient healthcare system in Spain, where healthcare spending represents 7.2% of GDP (EUR 2174 per capita), compared to 13.9% of GDP (EUR 10,348 per capita) in the US [11]. A clear example of this discrepancy is that our patients experienced an average hospital stay of 20 days, with an associated cost of EUR 18,118 per admission. In contrast, patients in the US had a significantly shorter average stay of 5 days, with a corresponding cost of $13,998 [12]. Despite these differences, life expectancy is higher in Spain (77 vs. 84 years) [13], suggesting a greater cost-effectiveness in the Spanish healthcare system. Notably, the health expenditure per capita in Spain is approximately 21% of that in the US [11], which could help explain the significant cost disparity observed between our study and the US-based research. It is also worth noting the comparatively healthier lifestyle in Spain, which is associated with greater life expectancy and may contribute to a reduced number of hospital admissions and, consequently, lower associated healthcare costs [13].

Although a limited number of patients were treated upfront with tagraxofusp, we were able to calculate the overall associated costs. These patients had marginally shorter lengths of stay than those observed in the overall study population, and their average reimbursement per inpatient hospitalization was also lower (Table S5). However, they spent a significant proportion of their remaining lifetimes hospitalized, similar to the overall cohort. The total reimbursement cost for patients receiving upfront tagraxofusp was slightly lower than in the overall cohort (EUR 96,683 vs. EUR 109,104), even though three out of six underwent alloHSCT, and this procedure is a procedure known to be a major driver of hospitalization-related costs. In contrast, analyzing only the complete CTH period, a 16% increase in total cost was observed in the tagraxofusp as first-line therapy subgroup compared to patients treated with conventional CHT at first line. However, these results should be interpreted with caution, as most patients in the tagraxofusp group were treated more recently, introducing a potential bias. Although it could be assumed that long-term survivors would contribute to increased overall costs and prolonged hospital stays, our findings revealed the opposite trend. This counterintuitive result may be explained by the fact that patients in the more recent period were more frequently treated with intensive regimens (including alloHSCT), whereas previously they would have been candidates for non-intensive therapies that typically did not require hospitalization. Additionally, the number of outpatient clinic visits and day hospital attendances was higher in the recent period, likely reflecting both the broader use of active treatment strategies and improvements in therapeutic options.

It is essential to acknowledge the limitations of our analysis, particularly due to its retrospective design and the relatively small sample size. A key limitation is the potential selection bias inherent to the retrospective design, given that patients diagnosed in earlier periods may not have complete HCRU records and thus may have been systematically excluded based on the study’s inclusion criteria. It should also be noted that data for this analysis were extracted in less than 20% of cases, introducing a potential collection bias. Nevertheless, our study offers valuable insights into the real-world economic impact of BPDCN management, providing a foundation for future research aimed at optimizing healthcare resource allocation in this rare and challenging disease. While acknowledging the limited sample size, we should be cautious regarding any potential HCRU and cost savings using tagraxofusp, and these potential savings should be contrasted with the list price of this agent as compared with classical CHT agents.

## 5. Conclusions

There is a significant consumption of economic and healthcare resources in patients with BPDCN undergoing active treatments, reflecting the complexity and aggressiveness of the disease. However, the emergence of novel therapies, such as CD123-targeted therapies, offers the potential to reduce HCRU by improving response rates and reducing relapses, while also potentially lowering indirect costs due to their reduced toxicity. Balancing the economic burden of these innovative treatments with their clinical benefits remains a challenge. Therefore, it is important to conduct comparative pharmacoeconomic studies across various treatment options for BPDCN to evaluate their cost-effectiveness and identify the patient subgroups that may derive the benefit from these new therapies.

## Figures and Tables

**Table 1:** Patient characteristics.

Characteristic	n = 38
Age at index date, years	
Median [IQR]	66 [18]
Mean (SD)	61 (16)
Men, number (%)	32 (84)
Status at the end of follow-up, number (%)	
Deceased	25 (66)
Alive	13 (34)
Type of scheme at first-line, number (%)	
Intensive	22 (58)
Non-intensive	16 (42)
First-line treatment received, number (%)	
AML-Like	12 (32)
Idarubicin + cytarabine (3 + 7)	7 (18)
FLAG-IDA	3 (8)
LDAC	1 (3)
FLUGA	1 (3)
ALL-Like	6 (16)
HYPERCVAD	6 (16)
Lymphoma-Like	6 (16)
CHOP	5 (13)
SMILE	1 (3)
Targeted therapies ^†^	14 (37)
Monoclonal antibodies	8 (21)
Tagraxofusp	6 (16)
Patients with at least one hospitalization at period, number (%)	
CHT	35 (92)
alloHSCT	18 (47)

^†^ Targeted therapies include anti-CD123 and anti-CD33, among others; *ALL*—acute lymphocytic leukemia; *alloHSCT—*allogeneic hematopoietic stem cell transplantation; *AML—*Acute Myeloid Leukemia; CHOP—cyclophosphamide/doxorubicin/vincristine/prednisone; *FLAG-IDA—*fludarabine/cytarabine/filgrastim/idarubicin; CHT—chemotherapy; *FLUGA—*fludarabine/cytarabine/filgrastim; *HYPERCVAD*—cyclophosphamide/vincristine/doxorubicin/dexamethasone; *IQR*—Interquartile Range; *LDAC*—low-dose cytarabine; *SD*—standard deviation; *SMILE*—dexamethasone/methotrexate/ifosfamide/L-asparaginase/etoposide.

**Table 2:** Mean and median healthcare resource utilization (HCRU) across the full patient lifetime, from diagnosis to death or last follow-up.

Healthcare Resource Unit	Inpatient Hospitalizations	External Consultation Visits	Day Hospital Visits	Overall
Per hospitalization				
Number of hospitalization or visits	187	1480	1100	NA
Mean (SD), median [IQR] length of stay per episode, days	20 (28) 11 [23]	1 (0)	1 (0)	NA
Mean (SD), median [IQR] reimbursement per hospitalization or visit, EUR	18,118 (19,345) 15,700 [20,392]	231 (0)	262 (0) †	NA
Per patient				
Mean (SD), median [IQR] number of stays	5 (3) 4 [5]	NA	NA	NA
Mean (SD), median [IQR] days of hospitalization	96 (100) 71 [87]	NA	NA	NA
Mean (SD), median [IQR] reimbursement, EUR	89,158 (77,408) 72,624 [69,089]	7439 (6371) 5062 [9359]	12,507 (15,424) 7271 [20,707]	109,104 (88,499) 93,085 [101,673]
Mean (SD), median [IQR] number RBC packages transfusion	21 (26) 12 [18]	NA	10 (15) 4 [8]	30 (36) 18 [33]
Mean (SD), median [IQR] number platelet transfusion	27 (41) 14 [26]	NA	11 (21) 2 [14]	38 (57) 15 [37]
Patients with ICU hospitalization, number (%)	9 (24)	NA	NA	NA
Mean (SD), median [IQR] days ICU hospitalization of patients admitted at ICU	10 (11) 5 [10]	NA	NA	10 (11) 5 [10]

^†^ EUR 691 after allogeneic hematopoietic stem cell transplantation (alloHSCT). ICU—intensive care unit; IQR—interquartile range; NA—not applicable; SD—standard deviation; RBC—red blood cells.

**Table 3:** Hospitalizations and reimbursement during the chemotherapy period (before the first allogeneic hematopoietic stem cell transplantation [alloHSCT] and in patients with no alloHSCT).

Healthcare Resource Unit	Inpatient Hospitalizations	External Consultation Visits	Day Hospital Visits
Per hospitalization			
Number of hospitalization or visits	128	723	676
Mean (SD), median [IQR] length of stay, days	16 (15) 10 [18]	1 (0)	1 (0)
Mean (SD), median [IQR] reimbursement, EUR	12,427 (7979) 9674 [10,085]	231 (0)	262 (0)
Per patient			
Number of patients	38	38	38
Mean (SD), median [IQR] number of stays	3 (2) 3 [4]	19 (16) 17 [19]	18 (23) 8 [23]
Mean (SD), median [IQR] days of hospitalization	54 (39) 56 [54]	NA	NA
Mean (SD), median [IQR] reimbursement, EUR	41,858 (27,038) 42,233 [39,465]	3634 (3115) 3247 [3629]	4793 (6195) 2291 [6198]

*IQR*—interquartile range; *NA*—not applicable; *SD*—standard deviation.

**Table 4:** Hospitalizations and reimbursement after allogeneic hematopoietic stem cell transplantation (alloHSCT).

Healthcare Resource Unit	Inpatient Hospitalizations	External Consultation Visits	Day Hospital Visits
Per hospitalization			
Number of hospitalization or visits	59	757	424
Mean (SD), median [IQR] length of stay, days	27 (44) 16 [26]	1 (0)	1 (0)
Mean (SD), median [IQR] reimbursement, EUR	30,464 (28,883) 27,017 [38,254]	231 (0)	691 (0)
Per patient			
Number of patients	18	18	18
Mean (SD), median [IQR] number of stays	3 (2) 3 [3]	33 (34) 19 [55]	24 (25) 17 [31]
Mean (SD), median [IQR] days of hospitalization	89 (92) 54 [50]	NA	NA
Mean (SD), median [IQR] reimbursement, EUR	99,855 (76,679) 72,990 [44,875]	16,287 (17,174) 11,754 [21,434]	6356 (6462) 3629 [10,505]

*IQR*—interquartile range; *NA*—not applicable; *SD*—standard deviation.

## Data Availability

The datasets generated and analyzed during the current study are property of the IISLAFE.

## Supplementary Materials

The following supporting information can be downloaded at: https://www.mdpi.com/article/10.3390/cancers17172844/s1. Table S1: Cost by DRG, and number of hospitalizations and patients according to DRG. Figure S1: Patients included in the HCRU study by year (n = 38). Table S2: First-line outcomes in the healthcare resource utilization (HCRU) cohort, stratified by treatment scheme and type, expressed as a number (%). Table S3: Hospitalizations and reimbursement among patients treated with tagraxofusp as a first-line therapy during the chemotherapy period. The overall average reimbursement was EUR 56,903 (SD EUR 13,947). Table S4: Hospitalizations and reimbursement during the chemotherapy period in patients not treated with tagraxofusp as a first-line therapy. The overall average reimbursement was EUR 49,044 (SD EUR 30,361). Table S5: Overall hospitalizations and healthcare reimbursement among patients treated with tagraxofusp as a first-line therapy. Figure S2: Reasons for inpatient hospitalization during the study period (n = 297). Reasons occurring four times or more are shown; all others are grouped together in ‘Other’ category. Patients could be hospitalized for more than one reason.

## Abbreviations

The following abbreviations are used in this manuscript:

ALL	Acute Lymphoblastic Leukemia
alloHSCT	Allogeneic Hematopoietic Stem Cell Transplantation
AML	Acute Myeloid Leukemia
BPDCN	Blastic Plasmacytoid Dendritic Cell Neoplasm
CD123	Cluster of Differentiation 123
CHT	Chemotherapy
CR	Complete Remission
CRi	Complete Remission with incomplete hematologic recovery
DRG	Diagnosis-Related Group
FLUGA	Fludarabine/Cytarabine/Filgrastim
FLAG-IDA	Fludarabine/Cytarabine/Filgrastim/Idarubicin
HCRU	Healthcare Resource Utilization
HYPERCVAD	Cyclophosphamide/Vincristine/Doxorubicin/Dexamethasone
ICU	Intensive Care Unit
IQR	Interquartile Range
LDAC	Low-Dose Cytarabine
MPAL	Mixed-Phenotype Acute Leukemia
NA	Not Applicable
OS	Overall Survival
PETHEMA	Programa Español de Tratamientos en Hematología
RBC	Red Blood Cells
SD	Standard Deviation
SMILE	Dexamethasone/Methotrexate/Ifosfamide/L-Asparaginase/Etoposide
