# Supplementary material for: Healthcare Resource Utilization and Treatment Costs for Blastic Plasmacytoid Dendritic Cell Neoplasm: A PETHEMA Study
Source: Cancers (Basel). 2025 Aug 29;17(17):2844. doi: 10.3390/cancers17172844 (PMC12427538; doi:10.3390/cancers17172844)

Table S1. Cost by DRG, and number of hospitalizations and patients according to DRG.

DRG code	DRG code abbreviated	DRG cost	Number of hospitalization per DRG (n=187)	Number of patients per DRG (n=36)
695- Chemotherapy for acute leukemia (minor severity)	695-Minor severity	€ 5,615	34	14
690- Acute leukemia (minor severity)	690-Minor severity	€ 6,625	48	25
690- Acute leukemia (moderate severity)	690-Moderate severity	€ 12,722	9	7
695- Chemotherapy for acute leukemia (major severity)	695-Major severity	€ 15,700	51	25
690- Acute leukemia (major severity)	690-Major severity	€ 27,017	19	15
690- Acute leukemia (extreme severity)	690-Extreme severity	€ 42,233	6	5
695- Chemotherapy for acute leukemia (extreme severity)	695-Extreme severity	€ 44,849	20	18

Figure S1. Patients included in the HCRU study by year (n=38).

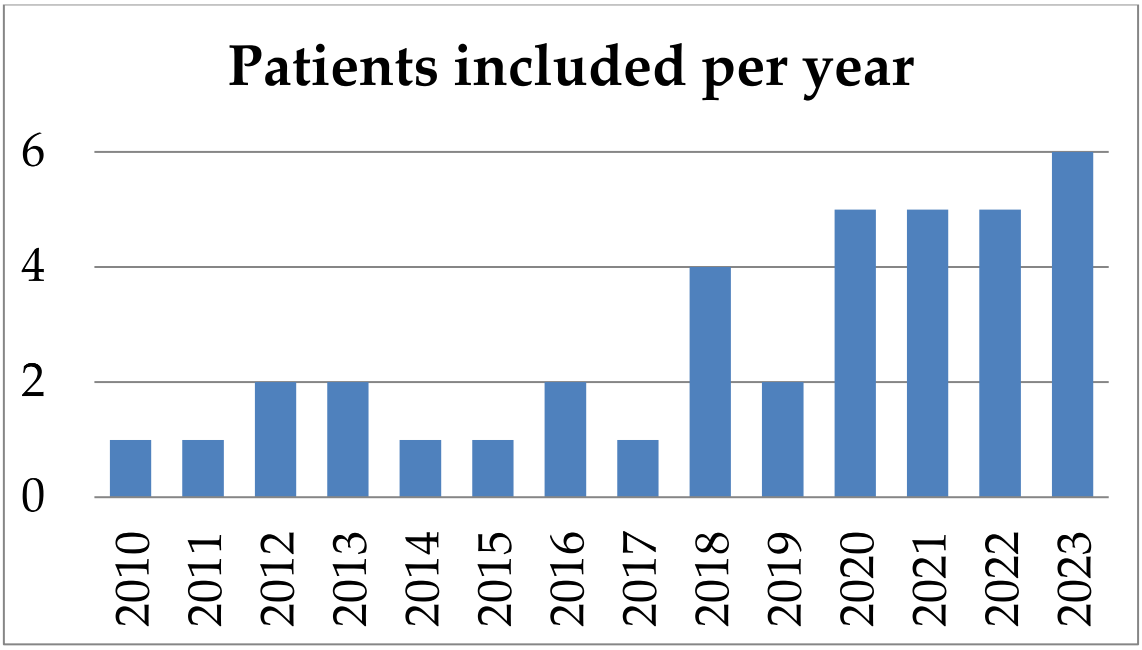

Table S2. First-line outcomes in the Healthcare Resource Utilization (HCRU) cohort, stratified by treatment scheme and type, expressed as number (%).

	**All patients**	**Treatment scheme**		**Treatment type**			
		**Intensive treatment**	**Non-intensive treatment**	**AML-Like**	**ALL-Like**	**Lymphoma -Like**	**Targeted therapies**
**Overall**	**38 (100)**	**22 (100)**	**16 (100)**	**12 (100)**	**6 (100)**	**6 (100)**	**14 (100)**
**CR**	**22 (57)**	15 (68)	7 (44)	5 (42)	6 (100)	4 (67)	7 (50)
**CRi**	**1 (3)**	0 (0)	1 (6)	0 (0)	0 (0)	0 (0)	1 (7)
**PR**	**1 (3)**	1 (5)	0 (0)	0 (0)	0 (0)	1 (17)	0 (0)
**ORR (CR+CRi+PR)**	**24 (63)**	16 (73)	8 (50)	5 (42)	6 (100)	5 (83)	8 (57)
**Resistance**	**11 (29)**	4 (18)	7 (44)	6 (50)	0 (0)	0 (0)	5 (36)
**Death**	**3 (8)**	2 (9)	1 (6)	1 (8)	0 (0)	1 (17)	1 (7)

ALL Acute Lymphocytic Leukemia; *AML* Acute Myeloid Leukemia; *CR* Complete Remission; *CRi* Complete Remission Incomplete; *ORR* Overall Response Rate; *PR* Partial Remission

Table S3. Hospitalizations and reimbursement among patients treated with tagraxofusp as a first-line therapy during the chemotherapy period. The overall average reimbursement was €56,903 (SD €13,947).

**Healthcare resource unit**	**Inpatient Hospitalizations**	**External Consultation Visits**	**Day Hospital Visits**
Per hospitalization			
Number of hospitalization or visits	26	106	75
Mean (SD), median [IQR] length of stay, days	12 (13)<br>6 [14]	1 (0)	1 (0)
Mean (SD), median [IQR] reimbursement, €	11,575 (7,391)<br>5,615 [10,085]	231 (0)	262 (0)
Per patient			
Number of patients	6	6	6
Mean (SD), median [IQR] number of stays	4 (3)<br>3 [6]	18 (26)<br>10 [14]	12 (29)<br>0 [3]
Mean (SD), median [IQR] days of hospitalization	53 (15)<br>56 [31]	NA	NA
Mean (SD), median [IQR] reimbursement, €	50,160 (15,261)<br>50,567 [22,373]	3,374 (4,878)<br>1,910 [2,674]	3,369 (7,862)<br>0 [808]

Table S4. Hospitalizations and reimbursement during the chemotherapy period in patients not treated with tagraxofusp as a first-line therapy. The overall average reimbursement was €49,044 (SD €30,361).

Healthcare resource unit	Inpatient Hospitalizations	External Consultation Visits	Day Hospital Visits
Per hospitalization			
Number of hospitalization or visits	102	617	601
Mean (SD), median [IQR] length of stay, days	17 (16)<br>11 [18]	1 (0)	1 (0)
Mean (SD), median [IQR] reimbursement, €	12,644 (8,142)<br>12,722 [9,075]	231 (0)	262 (0)
Per patient			
Number of patients	32	32	32
Mean (SD), median [IQR] number of stays	3 (2)<br>3 [4]	19 (15)<br>18 [18]	18 (22)<br>12 [23]
Mean (SD), median [IQR] days of hospitalization	54 (42)<br>56 [59]	NA	NA
Mean (SD), median [IQR] reimbursement, €	40,302 (28,620)<br>31,758 [48,794]	3,683 (2,780)<br>3,534 [3,438]	5,060 (5,948)<br>3,099 [6,198]

Table S5. Overall hospitalizations and healthcare reimbursement among patients treated with tagraxofusp as a first-line therapy.

Healthcare resource unit	Inpatient Hospitalizations	External Consultation Visits	Day Hospital Visits
Per hospitalization			
Number of hospitalization or visits	32	228	119
Mean (SD), median [IQR] length of stay, days	15 (15)<br>8 [24]	1 (0)	1 (0)
Mean (SD), median [IQR] reimbursement, €	15,185 (12,315)<br>15,700 [10,085]	231 (0)	262 (0)†
Per patient			
Number of patients	6	6	6
Mean (SD), median [IQR] number of stays	5 (3)<br>6 [6]	38 (47)<br>28 [32]	19 (46)<br>0 [5]
Mean (SD), median [IQR] days of hospitalization	80 (41)<br>85 [83]	NA	NA
Mean (SD), median [IQR] reimbursement, €	80,986 (35,101)<br>84,524 [68,605]	7,258 (8,947)<br>5,348 [6,112]	8,439 (19,618)<br>0 [2,191]

†€691 after allogeneic hematopoietic stem cell transplantation (alloHSCT). *IQR* Interquartile Range; *NA* not applicable; *SD* standard deviation.

Figure S2. Reasons for inpatient hospitalization during the study period (n = 297). Reasons occurring four times or more are shown; all others are grouped together in 'Other' category. Patients could be hospitalized for more than one reason.

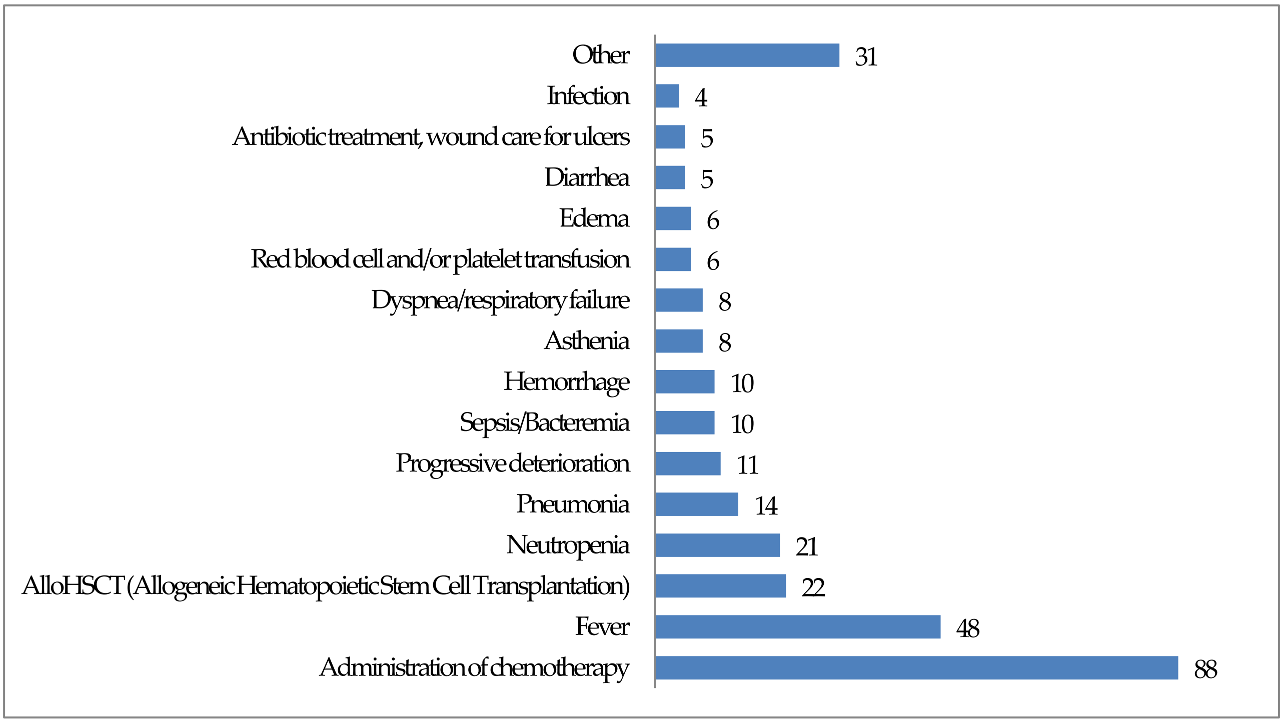

Supplement: Supplementary file 1 [file cancers-17-02844-s001.zip › cancers-3777429-supplementary.pdf]
